# Impact of genotype, body weight and sex on the prenatal muscle transcriptome of Iberian pigs

**DOI:** 10.1371/journal.pone.0227861

**Published:** 2020-01-28

**Authors:** Consolación García-Contreras, Ole Madsen, Martien A. M. Groenen, Adrián López-García, Marta Vázquez-Gómez, Susana Astiz, Yolanda Núñez, Rita Benítez, Almudena Fernández, Beatriz Isabel, Ana Isabel Rey, Antonio González-Bulnes, Cristina Óvilo

**Affiliations:** 1 Department of Animal Breeding, Instituto Nacional de Investigación y Tecnología Agraria y Alimentaria (INIA), Madrid, Spain; 2 Animal Breeding and Genomics, Wageningen University & Research, Wageningen, The Netherlands; 3 Faculty of Veterinary Medicine, Complutense University of Madrid (UCM), Madrid, Spain; 4 Department of Animal Reproduction, Instituto Nacional de Investigación y Tecnología Agraria y Alimentaria (INIA), Madrid, Spain; University of Florida, UNITED STATES

## Abstract

Growth is dependent on genotype and diet, even at early developmental stages. In this study, we investigated the effects of genotype, sex, and body weight on the fetal muscle transcriptome of purebred Iberian and crossbred Iberian x Large White pigs sharing the same uterine environment. RNA sequencing was performed on 16 purebred and crossbred fetuses with high body weight (340±14g and 415±14g, respectively) and 16 with low body weight (246±14g and 311±14g, respectively), on gestational day 77. Genotype had the greatest effect on gene expression, with 645 genes identified as differentially expressed (DE) between purebred and crossbred animals. Functional analysis showed differential regulation of pathways involved in energy and lipid metabolism, muscle development, and tissue disorders. In purebred animals, fetal body weight was associated with 35 DE genes involved in development, lipid metabolism and adipogenesis. In crossbred animals, fetal body weight was associated with 60 DE genes involved in muscle development, viability, and immunity. Interestingly, the results suggested an interaction genotype*weight for some DE genes. Fetal sex had only a modest effect on gene expression. This study allowed the identification of genes, metabolic pathways, biological functions and regulators related to fetal genotype, weight and sex, in animals sharing the same uterine environment. Our findings contribute to a better understanding of the molecular events that influence prenatal muscle development and highlight the complex interactions affecting transcriptional regulation during development.

## Introduction

Intensive swine production relies on commercial breeds highly selected for traits such as rapid growth or reproduction. However, there is also increasing interest in using traditional genotypes for producing high-quality, dry-cured meat products. Local pig breeds usually have not undergone intense genetic selection, are less productive than modern commercial breeds, and are traditionally raised in extensive production systems. Traditional swine usually feature small bodies, long productive cycles, slow growth, high food intake, and high fat deposition, so their meat differs from that of commercial breeds [1]. One of the most representative traditional breeds is the Iberian pig, which is farmed in Spain and Portugal.

Variation in prenatal weight even within the same litter can be a problem for animal viability and farm productivity, especially for breeds with a low uterine capacity such as the Iberian pig [2]. Deficiencies in placental growth or maternal nutrition can reduce the supply of nutrients and oxygen, leading to intrauterine growth restriction (IUGR) [3–5]. IUGR leads to offspring with low birth weight (LBW), which have reduced growth potential, high predisposition to adiposity [6–8], and undesirable changes in meat characteristics, such as greater toughness [9]. LBW offspring are also less healthy at birth and have a higher incidence of intestinal or immune disorders [10–12].

Postnatal performance and meat quality in swine are determined by both genetic and environmental factors, including the prenatal environment. In LBW piglets, epigenetic changes drive prenatal programming to increase survival, causing changes in adiposity as well as in carcass and meat traits [6–8]. Iberian pigs have developed a “thrifty genotype” to adapt to the harsh conditions of exploitation in traditional extensive breeding techniques [13]. These traits include longer production cycles [14]; increased energy accumulation in fat reserves; high levels of leptin circulating in plasma [15–17]; structural and functional variations in the leptin receptor (*LEPR*), which promotes leptin resistance [18]; and gene expression changes in the hypothalamus [19] and muscle [20, 21]. These “thrifty” metabolic traits mean that any alterations in prenatal environment may affect Iberian pigs more extensively than lean commercial breeds.

Prenatal muscle development is an important factor determining postnatal growth and carcass characteristics because the number of myofibers is fixed at birth, with postnatal muscle growth occurring only through myofiber hypertrophy [22–24]. In pigs, myogenesis occurs in two waves of myoblast proliferation and fusion: primary muscle fibers form from day 30 to 60 of embryonic development, while secondary fibers form from day 54 to 90 [25–27]. While the number and size of primary myotubes depends largely on genetic factors, secondary myotube formation is highly sensitive to maternal nutrition [28]. Prenatal intramuscular adipogenesis influences postnatal intramuscular fat accumulation, which is one of the most important parameters affecting meat quality. Iberian pigs have more oxidative muscle fibers, which is associated with more intramuscular fat and better meat quality than the meat of lean pig breeds such as Large White [29, 30].

Prenatal gene expression variations associated with muscle development and growth have been studied in many different pig breeds [27, 31–35]. In the Iberian pig, however, only postnatal transcriptomic analyses have been performed, which compared expression profiles between purebred Iberian pigs and crossbred Iberian x Duroc animals [20, 21, 36]). Characterizing gene expression patterns during prenatal muscle development enables a better understanding of the molecular basis of metabolic differences between traditional and commercial breeds, and between animals with low or high prenatal growth.

In this study, we aimed to determine the role of fetal genotype, weight, and sex on the transcriptomic profile of loin muscle. We studied two genotypes (purebred Iberian and crossbred Iberian x Large White), produced in one maternal environment (pure Iberian mothers) and selecting individuals with high body weight (HBW) and low body weight (LBW) to evaluate early changes in gene expression that may be associated with growth.

## Material and methods

### Ethics statement

All animal experiments were performed in compliance with the Spanish Policy for Animal Protection (RD1201/05), which meets the European Union Directive (86/609). All experimental procedures were approved by the INIA Ethics Committee for Animal Research (Institutional Animal Care and Use Committee or IACUC, CEEA 2013/036). Sows were housed at the INIA animal facilities, which meet local, national, and European requirements for Scientific Procedures Establishment.

### Animals and experimental design

Pure Iberian sows and boars of *Retinto* strain and Large White boars were employed. All sows and boars were genotyped by pyrosequencing as previously described [16] to confirm homozygosity for LEPRc.1987T (for the Iberian genotype) and LEPRc.1987C (for the Large White genotype). DNA for genotyping was extracted from frozen liver tissues using a commercial kit (Gentra Puregene Tissue Kit, Qiagen).

Ejaculate was collected from two purebred Large White and two Iberian boars and evaluated for semen quality (sperm concentration, morphology and motility). Ejaculates were mixed at a 1:1 ratio (Iberian : Large White) to obtain heterospermic semen, and aliquoted into 80 mL doses containing 6 x 10^9^ motile spermatozoa. Seven purebred Iberian sows underwent cycle synchronization with altrenogest (Regumate^®^, MSD, Boxmeer, The Netherlands) and were then inseminated with heterospermic semen. The sows were fed a standard grain-based diet (89.8% dry matter, 15.1% crude protein, 2.8% fat, 3.00 Mcal kg^-1^ ME) from the start of the experiment, adjusted to fulfil individual daily maintenance requirements, until gestational day 35. On gestational day 35, all sows were weighed and the amount of food offered to each sow was individually adjusted to 50% of their daily maintenance requirements. Our previous results have shown that this restricted diet affects fetal development and induces LBW [5]. Water was provided *ad libitum*.

On gestational day 77, corresponding to 70% of a 112-day total gestational period typical for this breed, all sows were euthanized by stunning and exsanguination, and the fetuses were collected. We chose this time point because it corresponds to the peak of secondary fiber formation [31, 37] and primary myotubes have already formed. A total of 51 fetuses were obtained. The position of the fetus in the genital tract was noted, and sex was determined by visual inspection. Several phenotypic traits were determined, including body weight, carcass weight, crown–rump length, occipital–nasal length, biparietal diameter, and thoracic and abdominal circumference. Tissues from the liver and *longissimus dorsi* muscle were collected from each fetus and stored at -80°C for genotyping and transcriptomic analysis. All 51 fetuses were genotyped, as previously done for the parents, for LEPRc.1987C/T [16]. Genotyping results indicated 27 animals were carriers of the homozygous TT genotype, corresponding to Iberian purebred animals (12 females and 15 males) and 24 were heterozygous CT animals, corresponding to the Iberian x Large White crossbred genotype (10 females and 14 males). The weight of the fetuses was employed to select 32 individuals phenotypically divergent for body weight for transcriptomic analysis (16 from each genotype and from each sex). The weight category was balanced across the individual progenies, i.e. each sow was similarly represented in each category.

### Analysis of plasma metabolites

Fetal blood samples were drawn from the heart and/or umbilical cord using heparinized syringes (Vacutainer Systems Europe, Meylan, France). The blood was centrifuged at 1500 *g* for 15 min immediately after recovery, and the plasma was stored in polypropylene vials at −20°C until analysis. Plasma glucose, fructosamine, triglycerides, total cholesterol, high-density lipoprotein cholesterol (HDL-c) and low-density lipoprotein cholesterol (LDL-c) were assayed using a Saturno 300 Plus analyzer (Crony Instruments s. r. l., Rome, Italy).

### Statistical analyses of phenotype and metabolic traits

The significance of effects of genotype, sex, and fetal weight on developmental and metabolic traits in the 32 animals selected for the transcriptome study was assessed using a mixed model that included the effects of genotype (purebred or crossbred), sex and weight category (HBW or LBW), with SAS 9.1 (SAS Inst. Inc., Cary, NC, USA). The fetus was considered the experimental unit for all variables and the mother was introduced into the model as a random effect. Results are expressed as mean ± SEM. Differences associated with *p* < 0.05 were considered significant.

### Transcriptome analysis

#### RNA extraction

Total RNA was extracted from 32 samples (50–100 mg) of *longissimus dorsi* muscle using the RiboPure RNA purification kit (Ambion, TX, USA) following the manufacturer’s protocols. RNA was quantified using a Nano-Drop-100 spectrophotometer (NanoDrop Technologies, Wilmington, DE, USA), and its quality was evaluated using the Agilent 2100 Bioanalyzer (Agilent Technologies, CA, USA). RNA Integrity Number (RIN) values in this study ranged from 7.5 to 9.8.

#### RNA library construction and sequencing

RNA libraries were made using the mRNA-Seq sample preparation kit (Illumina, USA) according to manufacturer protocols. Libraries were sequenced using a TruSeq SBS Kit v3-HS (Illumina) in paired-end un-stranded mode with a read length of 2 x 76 bp on a HiSeq2000 sequence analyzer (Illumina). Images from the instrument were processed using the manufacturer’s software to generate FASTQ sequence files.

#### Mapping and assembly

FastQC software (www.bioinformatics.babraham.ac.uk/projects/fastqc/) was used to determine the quality of the raw sequencing data. Trim Galore (version 0.4.1, https://www.bioinformatics.babraham.ac.uk/projects/trim_galore/) was used to remove sequencing adaptors and poly A and T tails (stringency of 6 bp, -s 6), keeping only paired-end reads where both pairs were longer than 40 bp. Reads were aligned against the Sscrofa11.1 genome using TopHat (version 2.1.0, (http://tophat.cbcb.umd.edu/) [38] with Bowtie2 (version 2.2.3, https://sourceforge.net/projects/bowtie-bio/files/bowtie2/) using default parameters. Transcripts were assembled and quantified in fragments per kilobase of transcript per million mapped fragments (FPKM) using Cufflinks (version 2.2.1, (http://cufflinks.cbcb.umd.edu/) [38]). Cuffmerge [39] was used to merge assembled transcripts from all samples with the reference transcripts, and Cuffquant [40] was used to pre-compute gene expression levels for each sample.

#### Differential expression analysis

The *Cuffdiff* tool in Cufflinks (version 2.2.1) was used to calculate expression values and perform differential expression analysis. Bias correction (option *-b*) was used to improve accuracy of transcript abundance estimates, and the “rescue method” for multi-reads (option *-u*) was used to weight read mapping more accurately.

Genes and new isoforms were filtered according to two criteria: minimum mean expression of > 0.5 FPKM in at least one group and a fold-change (FC) of ≥ 1.3 between different genotypes (purebred *vs*. crossbred), sexes (purebred_Females *vs*. purebred_Males and crossbred_Females *vs*. crossbred_Males), or body weights (purebred_LBW *vs*. purebred_HBW and crossbred_LBW *vs*. crossbred_HBW). Previous studies reported that this FC threshold improves results by eliminating background noise [41]. The false discovery rate (FDR) was calculated using the R package q-value [42]. Genes with a p-value and q-value ≤ 0.05 were considered differentially expressed (DE).

#### Functional analysis

DE genes were functionally analyzed using Ingenuity Pathways Analysis (IPA) software (Ingenuity Systems, www.ingenuity.com) to detect enriched pathways, biological functions, and potential regulators.

#### Reverse transcription-quantitative PCR (RT-qPCR)

Seven DE genes (*FOS*, *TRIM63*, *APOE*, *INSR*, *PPARG*, *PLIN1* and *ADIPOQ*) were examined by RT-qPCR of RNA from the same 32 fetuses that were subjected to RNA sequencing. Primers were designed against each gene, covering different exons, using Primer Select software (DNASTAR, Wisconsin, USA; **S1 Table**). The primers were designed to cover different exons to ensure amplification of cDNA. First-strand cDNA synthesis was performed following manufacturer protocols using Superscript II (Invitrogen, Life Technologies, Paisley, UK) and random hexamer primers in a total volume of 20 μL containing 1 μg of total RNA. Standard RT-PCR was first performed on cDNA to verify amplicon sizes. Expression was then quantified using SYBR Green mix (Roche, Basel, Switzerland) in a LightCycler480 (Roche) following manufacturer protocols. All samples were run in triplicate and dissociation curves were calculated for each reaction. PCR efficiency was estimated using a standard curve derived from serial cDNA dilutions (**S1 Table**). Expression was determined using the Cp method [43] and normalized against *GAPDH* and *ACTB* because analysis with Genorm software [44] identified them as the most stably expressed housekeeping genes. Normalized expression values were compared between groups using a *t* test. Pearson’s correlation was used to compare FPKM values from RNA sequencing with the results of qPCR.

## Results and discussion

The results obtained in the present study, evaluating effects of porcine fetal genotype, body-size and sex under the same maternal environment (same breed, parity number, management, nutritional status and litters), identified novel candidate genes and regulators potentially involved in the offspring phenotype and postnatal development. These findings provide new information on the functional genetics aspects of prenatal development and their potential relationship with productive parameters and meat quality in swine. The uniqueness of our study was that the changes observed were exclusively determined by fetal genotype, sex and weight, since the fetuses shared the same uterine environment (pure Iberian mothers), besides the extreme genotypes employed.

### Effects of fetal genotype, sex and weight on developmental traits

A total of 51 fetuses were obtained, of which 27 were purebred Iberian (12 females, 15 males) and 24 were crossbred Iberian x Large White (10 females, 14 males). Weights of the LBW and HBW groups differed 1.7 SD in purebred fetuses and 1.5 SD in crossbred fetuses.

The developmental and metabolic traits of purebred and crossbred fetuses selected for the transcriptome study are shown in **Table 1**. Crossbred fetuses were heavier (*p* < 0.0001) and more corpulent (higher biparietal diameter [*p* < 0.05] and trunk circumference [*p* < 0.01]), consistent with our previous observations in the whole dataset [45]. These differences may be explained by genetic differences in body size and weight between the breeds. It is well known that at postnatal stages, Iberian pigs are smaller than other commercial breeds, including Large White [46]. However, few studies have compared lean and obese pig breeds at prenatal stages, and we appear to be the only group to have compared these breeds in the same uterine environment [45].

**Table 1:** Effect of genotype, body weight and sex on developmental and metabolic traits of purebred Iberian and crossbred Iberian x Large White pig fetuses.

		Iberian					Iberian x Large White					p		
		All (n = 16)	LBW (n = 8)	HBW (n = 8)	F (n = 8)	M(n = 8)	All (n = 16)	LBW (n = 8)	HBW (n = 8)	F (n = 8)	M (n = 8)	Genotype	Weight	Sex
**Developmental traits **														
Body morphometry (cm)	**Body length**	22.9 ± 0.5	22.4 ± 0.7	23.4 ± 0.7	22.5 ± 0.7	23.3 ± 0.7	23.9 ± 0.5	23.8 ± 0.7	24.1 ± 0.7	23.8 ± 0.7	24.0 ± 0.7	0.130	0.355	0.482
	**Occipito-nasal length**	7.2 ± 0.6	7.3 ± 0.6	7.3 ± 0.6	6.9 ± 0.6	7.5 ± 0.6	7.3 ± 0.6	6.7 ± 0.6	7.8 ± 0.6	7.5 ± 0.6	7.1 ± 0.6	0.741	0.102	0.837
	**Biparietal diameter**	3.3 ± 0.1	3.3 ± 0.1	3.4 ± 0.1	3.2 ± 0.1	3.4 ± 0.1	3.5 ± 0.1	3.3 ± 0.1	3.7 ± 0.1	3.5 ± 0.1	3.5 ± 0.1	**0.042**	**0.039**	0.199
	**Trunk diameter**	3.3 ± 0.1	3.1 ± 0.2	3.5 ± 0.2	3.1 ± 0.2	3.6 ± 0.2	3.5 ± 0.1	3.3 ± 0.2	3.8 ± 0.2	3.6 ± 0.2	3.5 ± 0.2	0.193	**0.014**	0.219
	**Trunk circumference**	13.4 ± 0.3	12.6 ± 0.4	14.2 ± 0.4	12.8 ± 0.5	13.9 ± 0.5	14.7 ± 0.3	13.9 ± 0.4	15.5 ± 0.4	15.2 ± 0.5	14.2 ± 0.5	**0.005**	**0.001**	0.921
Body weight (g)	**Body weight**	293.1 ± 10.0	246.1 ± 14.5	340.1 ± 14.5	270.7 ± 25.4	315.0 ± 24.6	363.1 ± 10.0	311.3 ± 14.5	415.0 ± 14.5	365.0 ± 24.6	355.78 ± 24.06	**<0.001**	**<0.001**	0.240
	**Head weight**	75.0 ± 2.1	64.1 ± 3.0	85.9 ± 3.0	70.7 ± 5.1	79.5 ± 4.9	86.1 ± 2.1	77.6 ± 3.0	94.5 ± 3.0	86.9 ± 4.9	83.38 ± 4.82	**0.001**	**<0.001**	0.400
	**Carcass weight**	168.7 ± 8.2	139.0 ± 12.3	198.4 ± 12.3	155.7 ± 16.9	181.9 ± 16.4	217.7 ± 8.2	193.3 ± 12.3	242.1 ± 12.3	206.2 ± 16.5	226.3 ± 16.1	**<0.001**	**<0.001**	0.053
**Metabolic traits**														
Glucose metabolism (mg/dl)	**Glucose**	10.8 ± 3.5	13.3 ± 4.9	8.7 ± 5.3	9.3 ± 6.3	8.5 ± 5.5	21.1 ± 3.8	18.9 ± 4.9	25.6 ± 6.3	8.5 ± 5.9	33.9 ± 5.6	0.057	0.970	0.096
	**Fructosamine**	113.1 ± 6.1	105.1 ± 7.6	121.3 ± 6.9	114.3 ± 7.6	112.4 ± 7.3	122.6 ± 6.0	112.0 ± 7.1	133.4 ± 6.9	123.7 ± 7.2	118.1 ± 7.7	0.073	**0.001**	0.423
Lipid metabolism (mg/dl)	**Triglycerides**	22.6 ± 1.7	24.6 ± 2.6	20.6 ± 2.4	19.4 ± 2.1	25.5 ± 2.1	24.2 ± 1.7	26.6 ± 2.4	21.7 ± 2.6	24.5 ± 2.1	23.6 ± 2.2	0.511	0.084	0.222
	**Total cholesterol**	55.6 ± 4.2	53.4 ± 5.3	58.4 ± 4.7	58.4 ± 5.5	52.5 ± 5.2	43.4 ± 4.2	37.1 ± 4.7	49.5 ± 5.2	42.4 ± 5.3	41.7 ± 5.1	**0.008**	**0.045**	0.339
	**HDL-c**	14.3 ± 1.5	13.9 ± 1.7	14.7 ± 1.6	14.9 ± 1.7	13.7 ± 1.6	12.2 ± 1.5	10.9 ± 1.6	13.4 ± 1.6	12.4 ± 1.6	11.7 ± 1.6	0.080	0.125	0.349
	**LDL-c**	39.7 ± 2.0	35.7 ± 3.0	43.8 ± 2.8	39.9 ± 3.4	39.6 ± 3.2	29.7 ± 2.1	25.7 ± 2.7	33.8 ± 2.9	28.4 ± 3.3	30.87 ± 3.15	**0.003**	**0.017**	0.696

All data except p values are mean ± SEM.

Abbreviations: F, female fetuses; HBW, high body weight fetuses; HDL-c, high density lipoprotein cholesterol; LBW, low body weight fetuses; LDL-c, low density lipoprotein cholesterol; M, male fetuses

Fetal sex did not have any significant effect on developmental traits, although there was a trend for higher carcass weight in males than females, independent of genotype. In other words, development at this stage was affected mainly by fetal genotype, with no major sex-specific effects. Previous studies at prenatal and postnatal stages have shown that males are heavier than females when mothers are undernourished [47, 48].

### Effects of fetal genotype, sex, and weight on metabolic traits

Iberian fetuses had higher levels of total cholesterol (*p* < 0.01) and LDL-cholesterol (*p* < 0.005, **Table 1**) than crossbred fetuses, in agreement with previous studies [20, 49]. These results demonstrate that Iberian pigs have altered lipid metabolism and support the hypothesis that metabolic differences between fatty and lean breeds are established in the early prenatal stages. There was also a trend for higher plasma glucose (*p* = 0.057) and fructosamine (*p* = 0.073) in crossbred than Iberian fetuses. Glucose levels serve as an index of glycemia at the moment of sampling and is generally considered the main energy source for developing fetuses, while fructosamine levels are representative of average glucose values over the past several days [50].

Body weight also had a strong effect on metabolic profiles. In both genotypes, HBW fetuses had higher levels of fructosamine (*p* < 0.005), total cholesterol (*p* < 0.05), and LDL-cholesterol (*p* < 0.05) than LBW fetuses. There was also a trend towards higher triglyceride levels (*p* = 0.08) in HBW fetuses than LBW fetuses. These results demonstrate the importance of cholesterol and glucose availability on fetal development [45]. Fetal sex did not have any significant effects on metabolic traits.

### Transcriptomic analysis

Out of the 51 fetuses obtained, 32 (16 purebred and 16 crossbred fetuses) were selected for transcriptomic analysis. Each group contained 8 females (4 with HBW, and 4 with LBW) and 8 males (4 with HBW, and 4 with LBW). An average of 27 million paired-end RNA-Seq reads was obtained per sample, and an average of 93% of the quality-filtered reads were mapped and assembled to the porcine reference genome (Scrofa 11.1). The sequencing data has been deposited in GEO database, with accession number GSE140460.

**Fig 1:**
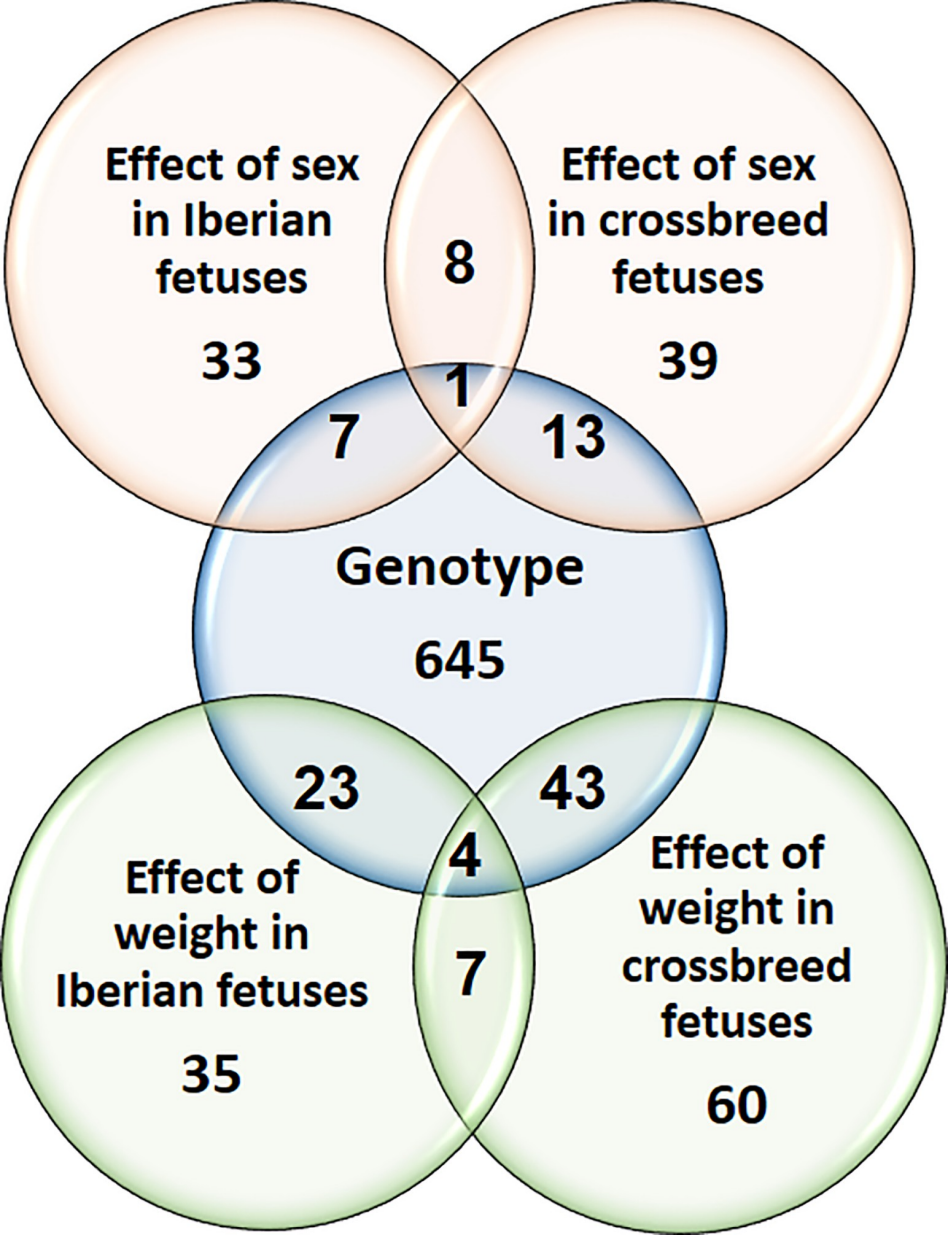
Venn diagram showing the total number of differentially expressed (DE) genes in all comparisons.

DE genes are summarized in **Fig 1**. To validate the RNA sequencing results, we measured the expression of 7 DE genes (*FOS*, *TRIM63*, *APOE*, *INSR*, *PPARG*, *PLIN1* and *ADIPOQ*) by qPCR (**S2 Table**). Differential expression was validated for five of the seven genes (*APOE*, *PPARG*, *ADIPOQ*, *PLIN1* and *TRIM63*), and the expression of all 7 genes correlated closely with RNA sequencing data (*r*^2^ = 0.87–0.99 and *p* < 0.0001 for all genes).

#### Effects of genotype on muscle transcriptome

After correction for multiple testing (*q*-value < 0.05), a total of 645 DE genes between Iberian and crossbred fetuses were identified for which FC > 1.3 (**S3 Table**). Of these, 185 were upregulated in Iberian fetuses (FC = 1.3–163) and 460 were upregulated in crossbred fetuses (FC = 1.3–799).

The gene with the largest difference in expression was *TBL1X* (ENSSSCG00000038226), which showed 799-fold higher expression in crossbred than Iberian fetuses, followed by *IBSP*, which showed 163-fold higher expression in Iberian than crossbred fetuses. *TBL1X* has regulatory roles in signal transduction, RNA processing, gene regulation, vesicular trafficking, cytoskeletal assembly and may play a role in the control of cell differentiation. It is one of the core proteins of NCoR transcriptional repressor complex [51] which has been shown to be a major corepressor complex for the nuclear receptor family of transcription factors. Deletion of individual components of the NCoR complex results in an increase in liver triglycerides, highlighting their importance in the regulation of metabolic gene transcription. Crossbred fetuses showed upregulation of candidate genes related to muscle development (*RNF19B*, *FOXO1*, *FOXO3*, *FOXO6 COL11A2*, *MYOD*, *IGF-I*, *FOS*), protein catabolism *(TRIM63)*, and regulation of energy balance and lipid metabolism (*INSRR*, *APOD*, *UCP3* or *LEPR*).

Due to the importance of muscle development in the pork industry, many porcine studies have focused on identifying myogenic regulatory factors [34, 52–53]. Among the genes differentially expressed in our samples, those related to myogenesis pathways were upregulated in crossbred fetuses. For instance, significant roles have been proposed for *RNF19B*, which has been linked to muscle response during stabilized weight loss, or *FOXO3* which is a transcription factor with important roles in muscle hypertrophy and atrophy [54]. We also observed upregulation of *INSR* and its paralog *INSRR*, which are related to energy balance and metabolism. *INSR* encodes the insulin receptor, which binds insulin and insulin growth factor (IGF)-I and -II. Binding of insulin and IGF-I activates the PI3K/AKT pathway, which in turn regulates growth and muscle development [55]. These results support previous studies which showed altered regulation of genes involved in energy metabolism during myogenesis [56]. Moreover, *MYOD* and *IGF-I* genes are of special interest for muscle development because *IGF-I* signaling has an impact on *MYOD* and *MYOF5* regulation, which are strongly involved in the myogenesis process. Our results suggest that myogenic routes are upregulated in crossbred fetuses during the prenatal period, leading to accelerated development and higher weight. In contrast, in our previous comparison of Iberian and crossbred Iberian x Duroc pigs during the postnatal period we found that some genes related to muscle development (*FOS*, *FOXO3A* or *TRIM63*) were upregulated in Iberian pigs [20, 21]. These two sets of results suggest that the same myogenic routes that are upregulated earlier in crossbred fetuses may subsequently be upregulated in purebred fetuses during the early postnatal period, possibly as an adaptive mechanism to enable compensatory growth and increase survival.

Iberian fetuses showed upregulation of many key genes related to lipid metabolism and adipogenesis including *PPARG*, *FABP4*, *CEBPA*, *PLIN1*, *DGAT2* and *ADIPOQ*. These genes were also identified as key regulatory factors controlling the expression differences observed in the functional analysis (see below). *ADIPOQ* encodes adiponectin, a cytokine secreted by adipose tissue that regulates energy homeostasis, glucose metabolism and lipid metabolism, and that is involved in preadipocyte differentiation [57]. Caloric restriction has been shown to increase circulating adiponectin in humans and animals [57]. This may be explained by the increased adipose tissue in bone marrow during caloric restriction, which contributes to an increase in circulating adiponectin. In our study, all sows were given a restricted diet, with all fetuses exposed to the same maternal environment. Thus, the increased *ADIPOQ* expression in Iberian fetuses could be related to their increased ability to accumulate adipose tissue in muscle [58].

Another key DE gene identified is *peroxisome proliferator-activated receptor γ* (*PPARG*), which is known as the master regulator of adipogenesis [59], and has also been identified as a regulator of gene expression differences in piglets of different genotypes [20]. *CEBPA* encodes CCAAT/enhancer-binding protein alpha, which is a key transcription factor involved in adipogenesis [60], and has been shown to modulate expression of many lipid homeostasis genes such as the gene coding for leptin (*LEP*). Leptin is a hormone that regulates many aspects of body weight homeostasis (energy balance, food intake and body weight) [61]. We observed upregulation of *LEP* in Iberian fetuses, while its receptor (*LEPR*) was downregulated. Leptin is secreted by adipocytes into the blood stream, and it interacts with its receptor in the hypothalamus, resulting in increased energy expenditure and physical activity as well as reduced food intake driving an adipose mass reduction. Higher levels of leptin in muscle are associated with increased lipid oxidation [62] and reduced triglycerides content. Nevertheless, obese genotypes typically display leptin resistance, with increased leptin levels but no lipopenic effects. We speculate that decreased expression of *LEPR* in Iberian fetuses causes a leptin-resistant phenotype, which has been observed at hypothalamic level [18]. Besides the downregulation of *LEPR* in pure Iberian pigs at hypothalamic level, in the present work we also observed downregulation of muscle *LEPR* in Iberian fetuses at prenatal stages, which had not been assessed before.

The gene *fatty acid binding protein 4 (FABP4)* is well known for its association with fat‐related traits in pigs [63–64]. FABP4 is secreted by adipocytes and recent studies suggest that it plays an important role in fatty acid storage as triacylglycerol in adipocytes, as well as in intramuscular fat development. Overall, Iberian fetuses show upregulation of many genes that control lipid metabolism and affect fatness parameters. This gene expression changes are likely to be associated with the increased serum total cholesterol and LDL-cholesterol that we observed in Iberian fetuses.

**Fig 2:**
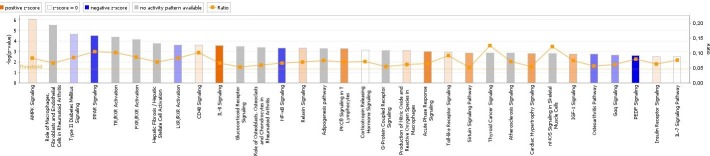
Top canonical pathways (*p* < 0.005) identified by functional analysis of DE genes in crossbred fetuses (orange bars) and Iberian fetuses (blue bars).

Functional analysis revealed a total of 117 significantly enriched pathways with *p* < 0.05 (**S4 Table**), including 32 with *p* < 0.005 (**Fig 2**). Many pathways were related to growth, development, adipogenesis, and energy and lipid metabolism.

Iberian fetuses showed downregulation of the signaling pathway mediated by AMP-activated protein kinase (AMPK, *p* = 8.24E-07). AMPK is a master metabolic regulator that controls glucose uptake, fatty acid oxidation, glycogen, cholesterol and protein synthesis, and induction of mitochondrial biogenesis [65]. AMPK plays a crucial role in the hypothalamus and peripheral tissues, where it controls dietary selection, first- and second-phase insulin secretion, lipid metabolism, and hepatic gluconeogenesis [66–67]. In fact, reduced AMPK activity in skeletal muscle and adipose tissue causes glucose intolerance and reduces exercise capacity, resulting in type 2 diabetes and obesity. Therefore, AMPK has been proposed as a promising drug target for obesity and type 2 diabetes. While leptin inhibits AMPK in the hypothalamus, it activates AMPK in peripheral tissues such as muscle [68], which implies that the overexpression of *LEP* and *ADIPOQ* that we observed in Iberian fetuses should lead to activation, rather than the observed downregulation of AMPK signaling in muscle. This finding may be explained by the reduction of *LEPR* expression in our obese individuals, which would impair leptin signaling and would therefore be a key factor in the inhibition of AMPK signaling in muscle besides its known determinant role at hypothalamic level in the Iberian obese animals [18].

Enrichment of pathways related to fat accumulation and deposition in Iberian fetuses may be related to their increased levels of total and LDL-cholesterol. Our results indicate an early upregulation of lipogenic pathways in Iberian fetuses. These results contrast with a previous study that found no difference in intramuscular fat between Iberian and Large White fetal pigs [45]. This may have been because pregnancy is a condition with high energy demand [69] and therefore most available lipids are used for fetal development rather than as an energy reservoir.

**Fig 3:**
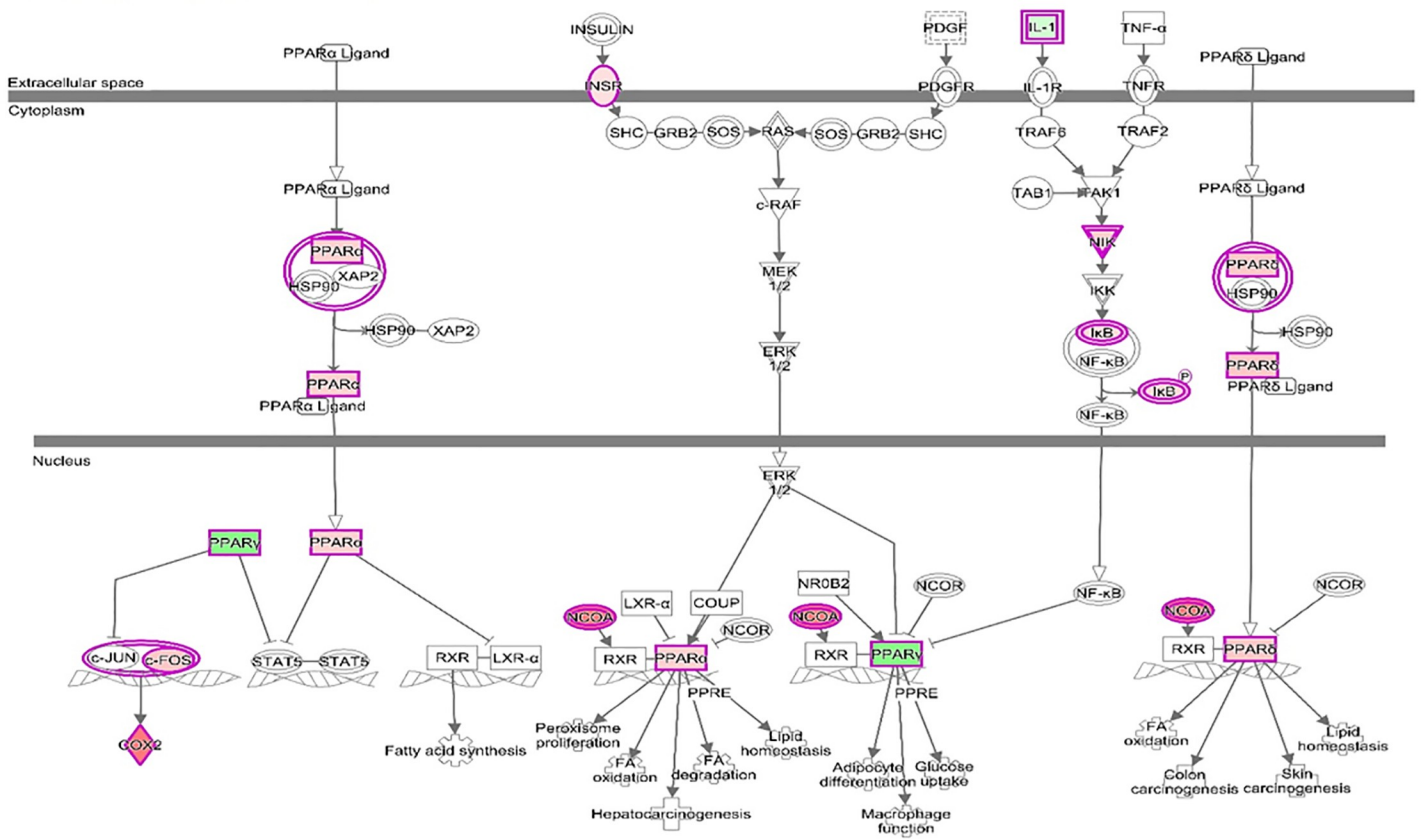
Enrichment of the PPAR signaling pathway in muscle from Iberian fetuses.

DE genes in our study were also involved in closely related key pathways such as *PPAR Signaling* (*p* = 3.19E-05, **Fig 3**), *Insulin Receptor Signaling* (*p* = 3.06E-03) and *Adipogenesis* (*p* = 5.57E-04). A previous study comparing muscle mRNA expression in two pig breeds at the extremes for fatty acid composition also observed enrichment of pathways related with fat deposition [70]. Insulin is an anabolic hormone essential for maintenance of whole-body glucose homeostasis, growth and development. In particular, insulin has an important role in increasing lipid synthesis in liver and fat cells and controlling fatty acid release from triglycerides in fat and muscle; while *PPARG*, as explained before, controls adipogenesis and handles the triglycerides storage.

The *IGF1 Signaling* and *Growth Hormone (GH) signaling* (*p* = 9.13E-03) pathways were activated in crossbred fetuses. IGF1 is essential during prenatal development, while GH is crucial during postnatal growth [71]. Both IGF1 and GH regulate metabolic processes including protein synthesis and adipose tissue development [72]. Crossbred fetuses showed higher expression of genes involved in the *IGF1 Signaling* pathway (*FOS*, *FOXO1*, *FOXO3*, *IGF1R)* and the *GH signaling* pathway (*FOS*, *IGF1R*). These results demonstrate the close relationship between endocrine and metabolic pathways and myogenesis, and how alteration of these pathways affects fat and protein deposition during muscle development.

Functional analysis identified 500 significantly enriched biological functions and diseases (**S5 Table**) with a *p*-value < 0.0001. Of these, IPA was able to predict the direction of change (increased or decreased) in 28 biological functions. In support of our results above, biological functions and diseases such as *Obesity*, *Concentration of lipids*, *Glucose metabolism disorder*, *Insulin resistance or Dysglycemia* were enriched in Iberian fetuses. In contrast, crossbred fetuses showed enrichment of biological functions related to growth and development (**S5 Table**).

**Fig 4:**
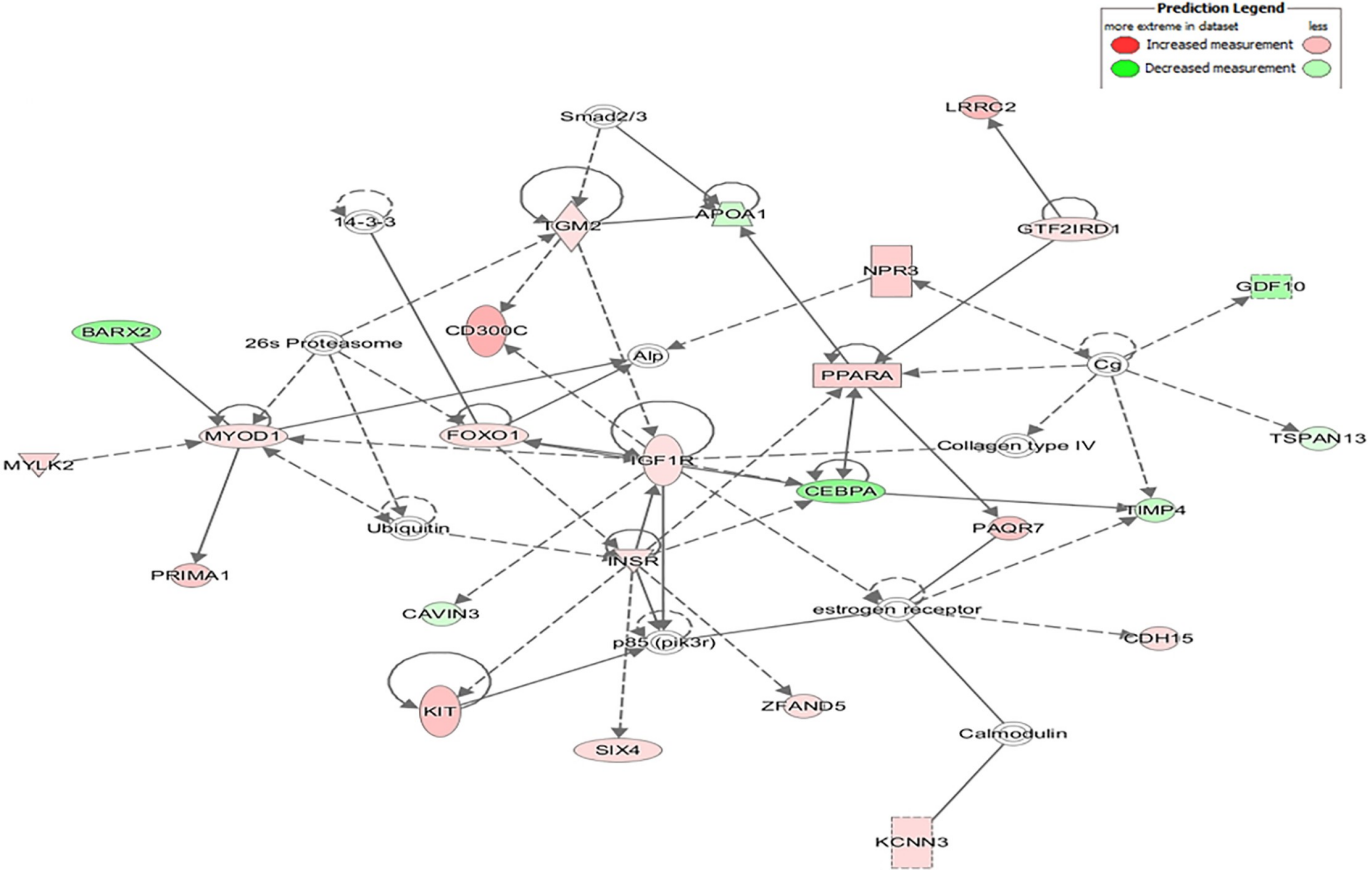
Functional network of *Metabolic Disease*, *Cellular Movement*, as well as *Skeletal and Muscular System Development and function* identified by analysis of DE genes in Iberian and crossbred fetuses. The intensity of colors represents the magnitude of differential expression, with red indicating genes upregulated in crossbred fetuses, while green indicates those upregulated in Iberian fetuses. Direct interactions are represented by solid lines, while indirect interactions are represented by dashed lines. The shapes of the nodes reflect the functional class of each gene product: transcriptional regulator (horizontal ellipse), transmembrane receptor (vertical ellipse), enzyme (vertical rhombus), cytokine/growth factor (square), kinase (inverted triangle) and complex/group/other (circle).

IPA identified several gene networks affected by fetal genotype, including the network *Metabolic disease*, *Cellular movement*, as well as *Skeletal and muscular system development and function* (**Fig 4**). The most central genes in this network (*FOXO1*, *IGF1R* and *INSR*) showed higher expression in crossbred fetuses, while *CEBPA* showed higher expression in Iberian fetuses. These results reinforce the importance of maintaining the correct balance between these genes in myogenesis and metabolic homeostasis.

**Fig 5:**
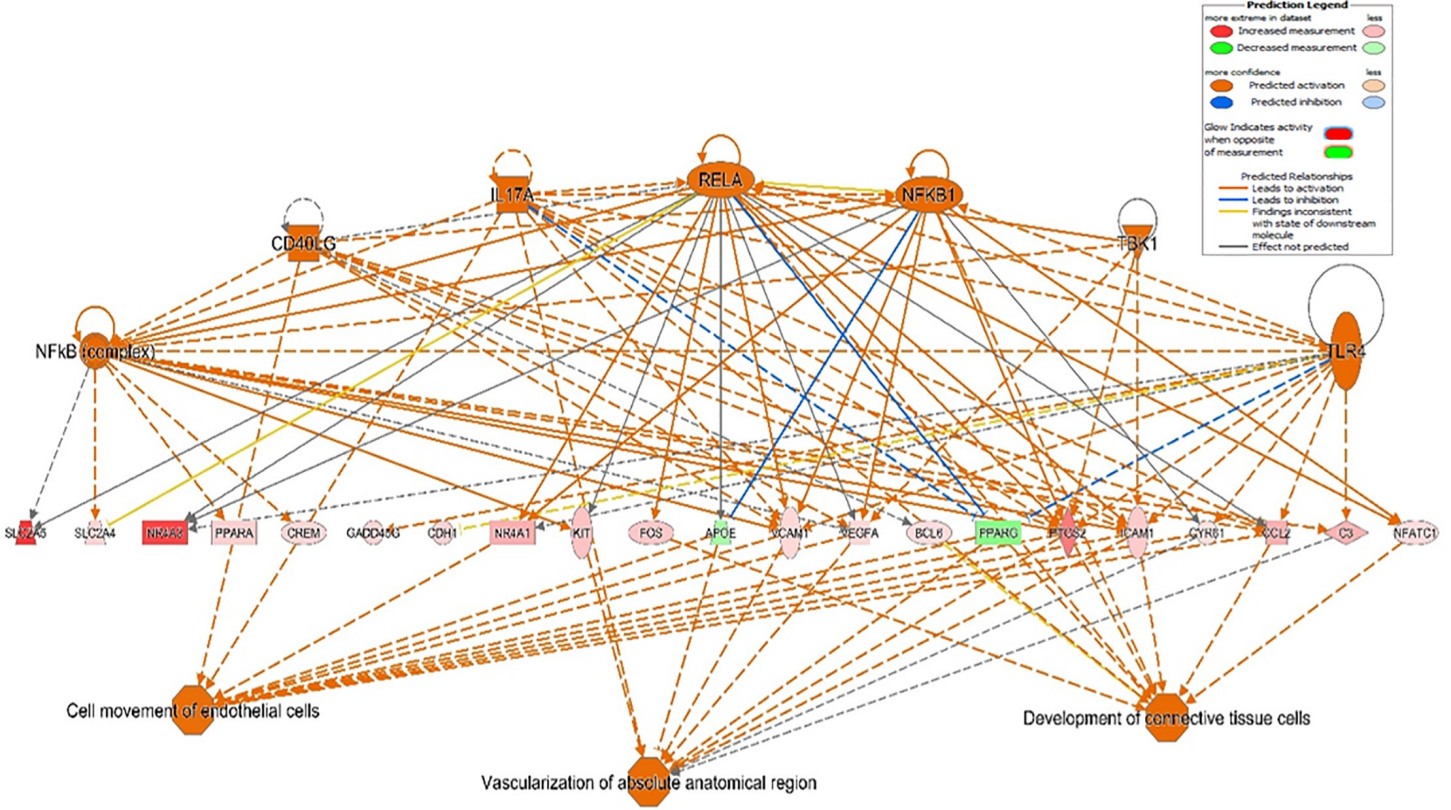
Top-scoring network of regulatory effects in crossbred fetuses. *Upper tier*: upstream regulators predicted to be activated (orange color). *Middle tier*: genes with altered expression in response to activation of upstream regulators (red = upregulation in crossbred; green = downregulation in crossbred). The shapes of the nodes reflect the functional class of each gene product: horizontal ellipse, transcriptional regulator; vertical ellipse, transmembrane receptor; vertical rhombus, enzyme; square, cytokine/growth factor; inverted triangle, kinase; and circle, complex/group/other. Direct interactions are represented by solid lines, while indirect interactions are represented by dashed lines. Orange lines lead to activation, while blue lines lead to inhibition. *Lower tier*: expected phenotypic consequences of gene expression changes based on data in the Ingenuity Knowledge Base (absolute z-score > 2 and *p*-value < 0.05). The octagonal symbol defines function.

To identify potential transcriptional regulators that may be behind gene expression changes, we screened *in silico* for potential regulatory factors. We identified 1208 potential upstream regulators (*p* = 2.15E-20 to 4.86E-02, **S6 Table**). Of these, 86 were DE between Iberian and crossbred fetuses: 29 were upregulated in Iberian fetuses (including *PPARG*, *APOE*, *CEBPA*, *LEP*, *ADIPOQ*, *PCK1* and *PLIN1*), while 57 were upregulated in crossbred fetuses (including *LEPR*, *FOXO3*, *INSR*, *IGF1R*, *VEGFA*, *MYOD1* and *TRIM63*). The top-scoring regulatory effects network is shown in **Fig 5**.

#### Effects of fetal weight on muscle transcriptome

We analyzed the effect of fetal weight separately in Iberian and crossbred fetuses due to the strong influence of genotype. The effect of fetal weight was slightly stronger in crossbred fetuses, with 60 DE genes between LBW and HBW individuals, while purebred LBW and HBW individuals differed in 35 DE genes (*q*-value < 0.05 and FC > 1.3, **S7 Table**). Of the 60 DE in crossbred fetuses, 52 were overexpressed in LBW fetuses (FC = 1.7–61) and 8 in HBW fetuses (FC = 2–13). Crossbred LBW fetuses showed upregulation of genes related to protein homodimerization activity (*ALAS2*), lipid and glucose metabolic processes (*APOD*), regulation of circadian rhythm (*NOCT*) and immunity (*GADD45A*, *GADD45B*, *IGFN1*), while HBW fetuses showed upregulation of genes related to skeletal system development (*COL9A2*) and regulation of bone mineralization (*MATN1*).

Apoliprotein D (*APOD*) is a member of the lipocalin superfamily containing lipid‐binding proteins such as fatty acid‐binding protein (*FABP*), retinol‐binding protein (*RBP*), and apolipoprotein M (*APOM*) [73]. *APOD* has multiple roles in lipid metabolism, embryogenesis, aging, and the central nervous system [74], and it is also involved in the conversion of HDL to LDL. Upregulation of *APOD* is consistent with our observation of higher plasma LDL levels in crossbred LBW fetuses than in HBW.

The circadian clock is important for regulating body temperature, blood pressure, and energy homeostasis [75]. Loss of circadian regulators such as *NOCT* has been reported to confer resistance to obesity induced by a high-fat diet. Upregulation of *NOCT* in LBW fetuses may therefore be associated with an increased predisposition to metabolic disease in adulthood, as we have observed in previous studies [48].

Growth arrest and DNA-damage-inducible protein 45 alpha gene (*GADD45A*) is a candidate regulator controlling Quantitative trait loci (QTL) for backfat thickness and intramuscular fat [76]. Both *GADD45A* and its paralog *GADD45B* are expressed in postmitotic adipocytes [77] and play a key role in active DNA demethylation during the differentiation of adipose-derived mesenchymal stem cells [78], as well as in the response to oxidative stress during pregnancy. Upregulation of *GADD45A* and *GADD45B* in LBW fetuses is consistent with IUGR, which causes oxidative stress and reduces antioxidant defense capacity [79]. Oxidative stress may also predispose animals to metabolic and cardiovascular diseases in adulthood.

Crossbred LBW fetuses showed lower expression of *COL9A2* and MATN1 compared to HBW fetuses, which are related to extracellular matrix structure and area of the *longissimus dorsi* muscle in pigs [80]. Genotype has previously been reported to affect *COL9A2* and *MATN1* expression in a study of purebred Iberian and crossbred Iberian x Duroc pigs in the postnatal stages [21]. We did not observe any effect of fetal genotype on *COL9A2* or *MATN1*, but fetal weight did have an effect on these genes in crossbred and Iberian fetuses (see below). Our results suggest that *COL9A2* and *MATN1* have important roles in muscle development at different developmental stages and in different genotypes, and that lower expression of these genes may indicate reduced muscle development in LBW fetuses.

Functional analysis of the 60 DE genes differing between crossbred LBW and HBW fetuses identified 32 enriched pathways (**S8 Table**). The most representative pathways were related to immunity (*GADD45 Signaling*, *IL-6 Signaling*, *IL-17A Signaling in fibroblasts or IL-12 Signaling*, and *Production in macrophages*). Although the direction of activation could not be predicted, all the DE genes in these pathways were overexpressed in LBW fetuses. Serum cytokines involved in innate immunity (TNF-α and IL-1β) have also been reported to be reduced in LBW piglets [81]. Women with IUGR pregnancies have higher levels of the pro-inflammatory cytokines IL-6, TNFα, and IL-12 than women with normal pregnancies.

A total of 500 diseases and biological functions were enriched in DE genes between LBW and HBW crossbred fetuses (*p* < 0.01, **S9 Table).** Of these, 82 were assigned an activation z-score and 5 were assigned a predicted activation state.

Functional analysis of DE genes showing higher expression in crossbred HBW fetuses showed enrichment of functions such as *Cell viability*, *Cell cycle progression* and *Size of body*, while crossbred LBW fetuses showed enrichment of functions such as *Inflammation of organ*, *Organismal death*, *Inflammatory response* and *Quantity of T lymphocytes*. A regulatory network related to *Skeletal and muscular system development and function* was also predicted. Skeletal and muscular system development (myogenesis) has been covered in several reviews [82–83]. Restricting maternal feeding is also known to decrease muscle fiber number and myonuclear number in rats [84] and guinea pigs [85]. In our study, all individuals were nutritionally restricted. Our results suggest that myogenic molecular processes are altered in LBW fetuses already during prenatal development.

Analysis of regulators identified 600 potential upstream regulators (*p* = 1.36E-07 to 4.92E-02), of which 7 were also DE. The most significant upstream regulators (*p* < 0.001) are shown in **Table 2**.

**Table 2:** Top potential upstream regulators of transcriptional differences between crossbred LBW and HBW fetuses.

Upstream Regulator	Expression Log Ratio	Molecule Type	Predicted Activation State	Activation z-score*	*p*-value of overlap	Target molecules in dataset
**CREM**		transcription regulator	Inhibited	-2.216	1.36E-07	CEBPB,FOS,GADD45B,NFIL3,NR4A1,PER1
**PDGF BB**		complex	Inhibited	-2.447	1.51E-07	ADM,CEBPB,FOS,GADD45A,NFIL3,NR4A1
**NR3C1**		ligand-dependent nuclear receptor		-0.152	3.77E-07	ADM,CEBPB,FOS,GADD45A,GADD45B,ISG15,NFIL3,PER1, PIM3, VCAM1
**Akt**		group	Inhibited	-2.216	4.79E-07	ADM,ANKRD1,CEBPB,FOS,NFIL3,VCAM1
**IKBKB**		kinase		-1.964	5.21E-07	ANKRD1,CEBPB,FOS,ISG15,MGP,NOCT,VCAM1
**CREB1**		transcription regulator	Inhibited	-2.401	8.04E-07	CEBPB,FOS,GADD45B,NFIL3,NR4A1,PER1
**RELA**		transcription regulator		-1.698	1.06E-06	ANKRD1,CEBPB,FOS,ISG15,NR4A1,TUBB3,VCAM1
**IL1B**		cytokine	Inhibited	-2.113	1.61E-06	CEBPB,FOS,ISG15,NFIL3,NOCT,NR4A1,STMN2,VCAM1
**Pkc(s)**		group		-0.73	5.59E-06	ADM,FOS,GADD45A,GADD45B,PER1
**IKBKG**		kinase	Inhibited	-2.177	6.29E-06	CEBPB,FOS,ISG15,MGP,NOCT
**ATF4**		transcription regulator	Inhibited	-2.2	7.06E-06	ANKRD1,CEBPB,EIF4EBP1,GADD45A,NUPR1
**UCP1**		transporter	Inhibited	-2.219	7.34E-06	ANKRD1,EIF4EBP1,GADD45A,MYH4,NUPR1
**TNF**		cytokine	Inhibited	-2.533	8.54E-06	ADM,CEBPB,FOS,GADD45A,ISG15,MAFF,MGP,NOCT,THBS2, VCAM1
**CHUK**		kinase	Inhibited	-2.213	1.09E-05	CEBPB,FOS,ISG15,MGP,NOCT
**CEBPB**	-1.146	transcription regulator			2.81E-05	CEBPB,FOS,GADD45A,HBB,NUPR1,TUBB3
**VCAN**		other		1.982	1.13E-04	ADM,C4A/C4B,CEBPB,VCAM1
**CTNNB1**		transcription regulator	Inhibited	-2.219	1.14E-04	APOD,C2orf40,F13A1,GADD45B,NR4A1,PRSS35,VCAM1
**IGF1**		growth factor		-0.737	1.22E-04	ADM,FOS,GADD45A,NR4A1,TUBB3
**IL17A**		cytokine		-1.96	1.45E-04	CEBPB,GADD45A,ISG15,VCAM1
**STAT3**		transcription regulator		-0.651	1.54E-04	ADM,ALAS2,CEBPB,FOS,GADD45A,ISG15
**AHR**		ligand-dependent nuclear receptor		0.988	3.48E-04	ADM,COL11A1,FOS,GADD45A,ITGBL1
**SMARCA4**		transcription regulator		0.371	7.06E-04	FOS,GADD45A,GADD45B,HBB,MAFF,MGP
**TREM1**		transmembrane receptor		-1	7.10E-04	CEBPB,GADD45B,ISG15,MAFF
**ADM**	-1.410	other			7.20E-04	ADM,FOS
**CG**		complex		-1.236	7.25E-04	ADM,CEBPB,COL11A1,NR4A1,STC1
**NFIL3**	-0.988	transcription regulator			7.99E-04	GADD45A,GADD45B
**PRL**		cytokine		0	9.05E-04	ISG15,MGP,NUPR1,PIM3
**MAPK1**		kinase		1	2.61E-03	FOS,HBB,ISG15,NUPR1
**IL4**		cytokine		-0.382	2.75E-03	FOS,ISG15,NFIL3,NOCT,VCAM1
**MYC**		transcription regulator		0.64	2.80E-03	ADM,GADD45A,GADD45B,MGP,THBS2,VCAM1
**FOXO1**		transcription regulator		-1.969	4.10E-03	FOS,GADD45A,GADD45B,VCAM1
**FOS**	-1.029	transcription regulator			4.83E-03	FOS,NFIL3,NR4A1,VCAM1
**CEBPA**		transcription regulator		0.152	5.60E-03	CEBPB,FOS,ISG15,NFIL3
**TGFB1**		growth factor		-1.387	8.87E-03	ADM,ANKRD1,FOS,ITGBL1,NUPR1
**PPARA**		ligand-dependent nuclear receptor		0.152	1.01E-02	CEBPB,FOS,PAQR7,VCAM1
**HBB**	-1.914	transporter			1.87E-02	VCAM1
**IFNG**		cytokine	Inhibited	-2.099	2.04E-02	ADM,C4A/C4B,CEBPB,ISG15,VCAM1
**NUPR1**	-1.165	transcription regulator	Inhibited	-2	2.09E-02	ADM,CEBPB,GADD45A,NFIL3
**NR4A1**	-1.625	ligand-dependent nuclear receptor			2.24E-02	ADM,APOD

HBW, high body weight fetus; LBW, low body weight fetus

*Indicates the activation state of predicted transcriptional regulators based on experimentally observed gene expression and the literature. It allows prediction of the direction of regulation (i.e. activating or inhibiting).

**Fig 6:**
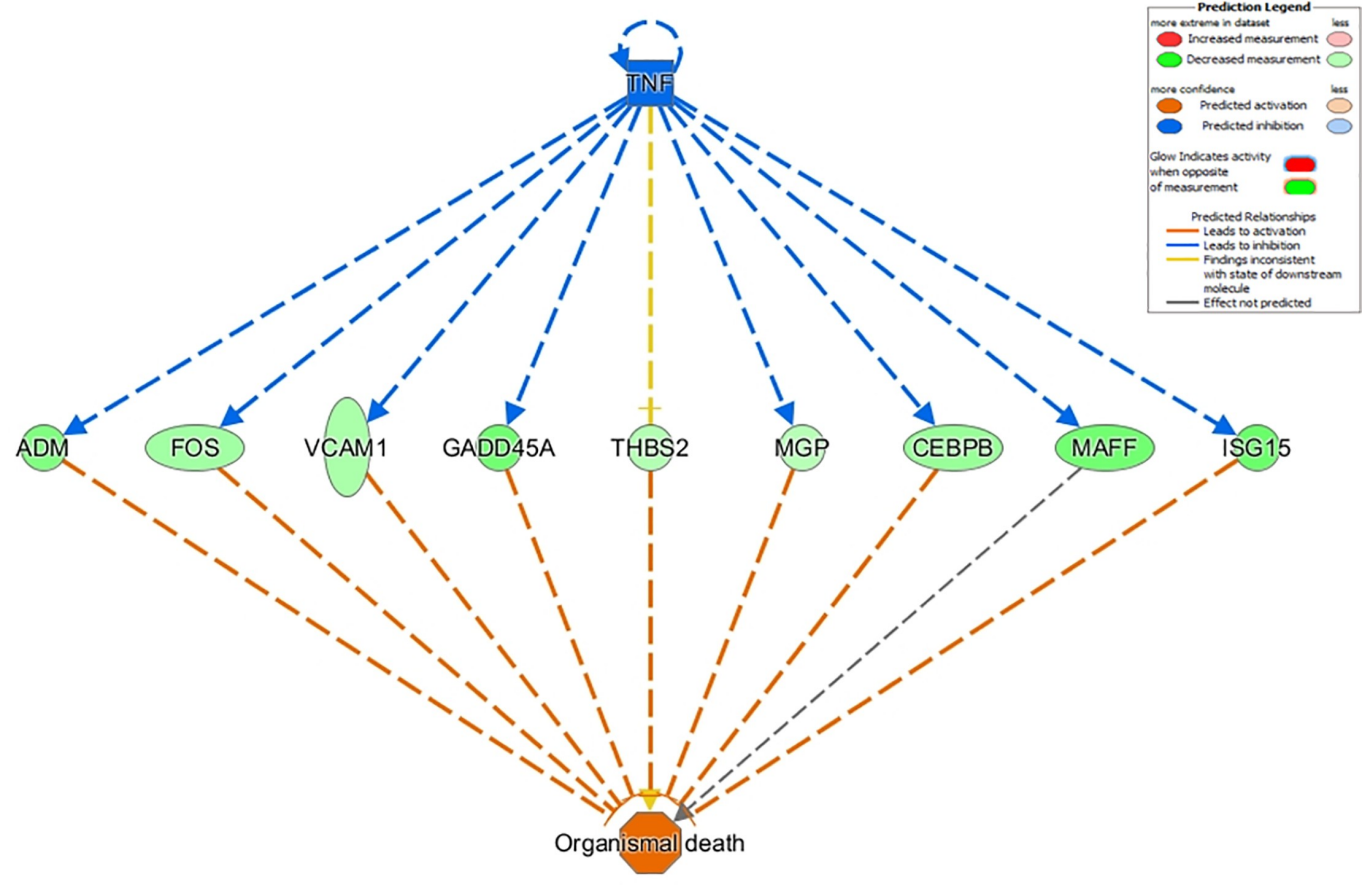
Top-scoring regulator effects network activated in crossbred LBW fetuses. *Upper tier*: one upstream regulator is predicted to be activated (blue). *Middle tier*: gene expression changes in response to activation of upstream regulators (green = upregulated in small fetuses). The shapes of the nodes reflect the functional class of each gene product: horizontal ellipse, transcriptional regulator; vertical ellipse, transmembrane receptor; and circle, complex/group/other. Direct interactions are represented by solid lines, while indirect interactions are represented by dashed lines. Orange lines lead to activation. *Lower tier*: expected phenotypic consequences of gene expression changes based on data in the Ingenuity Knowledge Base (absolute z-score > 2 and *p* < 0.05). The octagonal symbol defines function.

In crossbred LBW fetuses, regulators related to low-grade inflammation (such as *IL1B* and *TNF*) were activated (**Fig 6**), while regulators related to cell growth and differentiation (such as *VCAN* or *AHR*) were inhibited. These observations are consistent with the developmental phenotypes in LBW and HBW fetuses.

Fetal body weight had a smaller effect on gene expression in Iberian fetuses (35 DE genes) than crossbred fetuses (60 DE genes). In the Iberian genotype, there were more upregulated genes in HBW fetuses (32 DE genes) than in LBW fetuses (3 DE genes), while the opposite was observed in the crossbreed genotype (8 *vs*. 52 DE genes). When we compared the weight effect on the transcriptome we detected only 7 DE genes in common between the Iberian and crossbreed genotypes. Of these, 4 genes (*FOS*, *MGP*, *AHSP*, *SLC4A1*) showed evidences of genotype*weight interaction effects because they were upregulated in LBW fetuses for the crossbreed genotype, but in the Iberian genotype they were upregulated in HBW fetuses. These results may reflect the similarity in body weight and length between Iberian HBW and crossbred LBW fetuses in terms of phenotypic traits, which are phenotypically the closest groups with regards to several morphometry data **(Table 1**).

In addition to downregulating genes related to growth (*COL9A2*, *MATN1*, and *MATN4*), Iberian LBW fetuses also showed reduced expression of *FOS*, which is a regulator of cell proliferation, differentiation, and transformation and is related to diverse stimulus response [86]; and reduced expression of genes involved in carbohydrate and lipid metabolic processes or adipogenesis (*PLIN1*, *CIDEC*, *LPL*, *DGAT2*, *CEBPA*, *PCK1* or *ADIPOQ*). Expression of these genes was also affected by fetal genotype, with higher expression in Iberian than crossbred fetuses, as explained before (**S2 Table**). This increased expression may have contributed to more reliable measurement of gene expression in Iberian fetuses thus allowing the detection of DE between Iberian HBW and LBW fetuses.

Among these DE genes, downregulated in Iberian LBW animals, Perilipin-1 (*PLIN1*) is a key gene for porcine adipogenesis, and is closely related to the accumulation of intramuscular fat [87]. LBW Iberian fetuses also exhibited downregulation of *CIDEC*, which has been linked to adipocyte differentiation and metabolic disorders in humans [88], and which downregulates AMPKα and promotes adipogenesis [89]. Lipoprotein lipase (*LPL*) was also downregulated in LBW Iberian fetuses. Insulin is a well-known regulator of *LPL* [90], and *LPL* activity and triglyceride levels are related and are linked with the risk of cardiovascular and cerebrovascular diseases [91]. In agreement with these findings, we found that LBW Iberian fetuses had reduced total cholesterol, LDL-cholesterol and fructosamine, and a non-significant trend towards increased triglycerides (*p* = 0.08). Previous studies reported that fetal genotype and weight did not affect intramuscular fat [45], but this may have been due to the early stage of fetal development assayed. Studies from our group have found that Iberian piglets have higher intramuscular fat than crossbred Duroc x Iberian piglets at weaning [20, 36], and that HBW Duroc x Iberian neonates have higher intramuscular fat than LBW neonates [48]. In agreement, our results suggest upregulation of adipogenesis pathways in Iberian HBW fetuses (see below).

**Fig 7:**
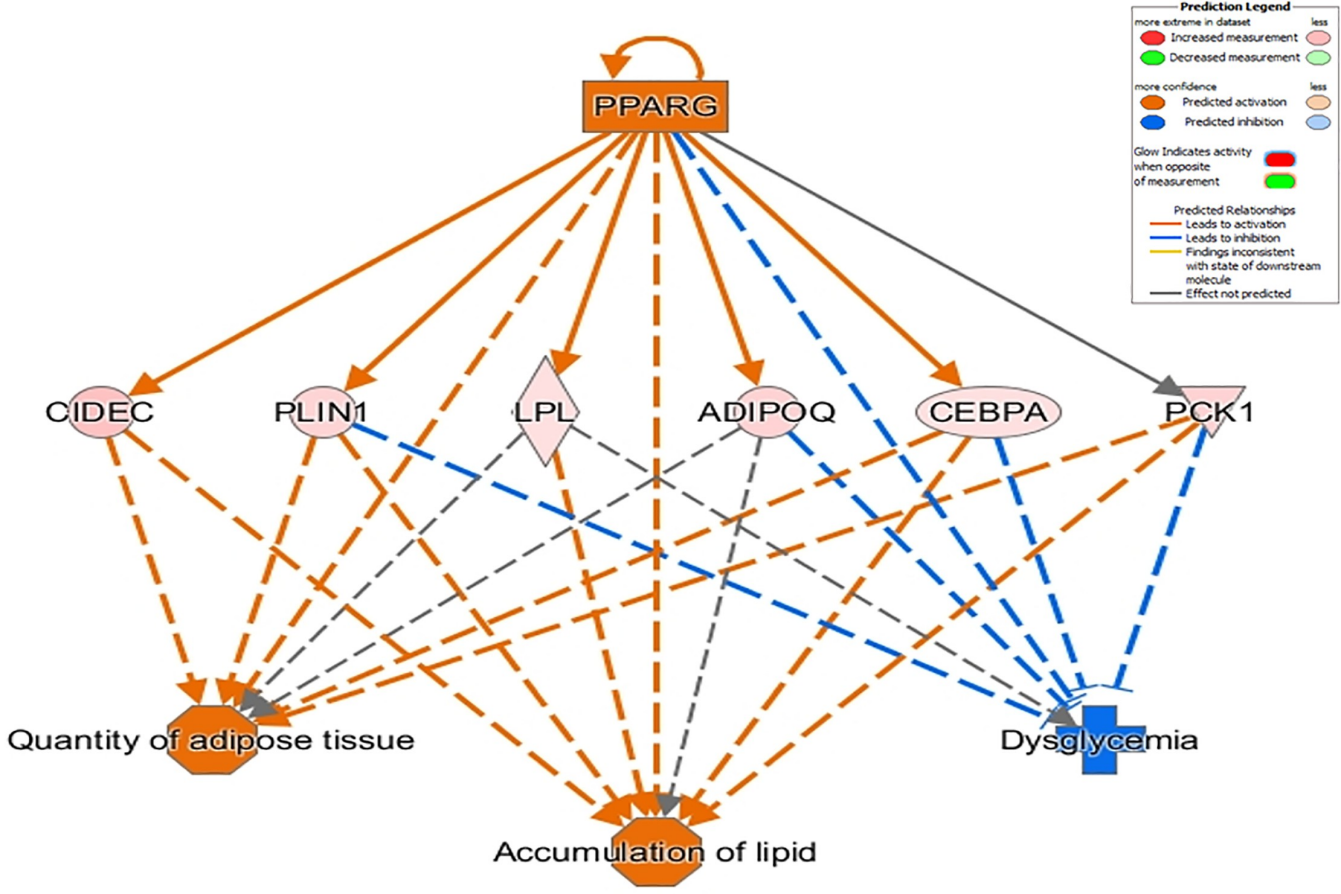
Top-scoring network of regulator effects activated in Iberian HBW fetuses. *Upper tier*: one upstream regulator is predicted to be activated (orange). *Middle tier*: gene expression changes in response to activation of upstream regulators (red = upregulated in large fetuses). The shapes of the nodes reflect the functional class of each gene product: horizontal ellipse, transcriptional regulator; vertical rhombus, enzyme; inverted triangle, kinase; and circle, complex/group/other. Direct interactions are represented by solid lines, while indirect interactions are represented by dashed lines. Orange lines lead to activation, while blue lines lead to inhibition. *Lower tier*: expected phenotypic consequences of gene expression changes based on data in the Ingenuity Knowledge Base (absolute z-score > 2 and p < 0.05). The octagonal symbol defines function.

Functional analysis of DE genes in LBW versus HBW Iberian fetuses identified 31 enriched pathways (**S8 Table**). In Iberian HBW fetuses, these included pathways related to lipid metabolism (*Adipogenesis*, *Glucocorticoid receptor signaling* and *Triacylglycerol degradation)*. Increased expression of fat deposition genes in Iberian pigs may affect detection of significant effects due to body weight. Functional analysis also identified 489 diseases and biological functions differing between HBW and LBW Iberian fetuses (*p* < 0.01), of which 32 were assigned an activation z-score and 6 were assigned a predicted activation state (**S10 Table**). The most significant disease detected was *Metabolic disease*, including *Dysglycemia*, *Glucose metabolism disorder* and *Insulin resistance*. All these were enriched in Iberian HBW fetuses, despite the fact that biochemical analysis did not reveal statistically significant differences in glucose between LBW and HBW fetuses. Biological functions such as *Size of body*, *Quantity of adipose tissue*, *Accumulation of lipid*, *Quantity of connective tissue* and *Synthesis of lipid* were enriched in Iberian HBW fetuses. Regulator analysis identified 623 potential upstream regulators (*p* = 5.93E-11 to 4.92E-02) that may explain the differences in expression patterns between LBW and HBW Iberian fetuses; of these, 7 were also DE genes. The main regulatory network identified involved *PPARG*, which was upregulated in Iberian HBW fetuses and related to functions such as *Accumulation of lipid*, *Dysglycemia* and *Quantity of adipose tissue* (**Fig 7**).

In summary, combined analysis of fetal weight in crossbred and Iberian fetuses indicates that crossbred HBW fetuses activate pathways involved in development, viability, survival and growth-related functions, while Iberian HBW fetuses activate lipid accumulation processes. This may indicate an early trend for myogenesis in crossbred HBW fetuses and adipogenesis in Iberian HBW fetuses. This would be consistent with differences between the two breeds in how much they tend to develop muscle or adipose tissue. In contrast, crossbred LBW fetuses show upregulation of the immune system and inflammatory response, while purebred LBW fetuses do not. We speculate that this finding may be related to a response of small crossbred fetuses to an Iberian maternal environment.

#### Effect of fetal sex on the muscle transcriptome

We evaluated the effect of fetal sex separately in each genotype. Only 39 DE genes were identified in crossbred fetuses (FC = 1.9–2,142) and 33 DE genes in Iberian fetuses (FC = 1.7–298, **S11 Table**). Eight DE genes were common to both genotypes. Interestingly, we observed potential qualitative interactions for genotype*sex in 5 of the 8 common DE genes (*COL9A2*, *COL9A1*, *LAT/SPNS1*, *CNMD* and *ENSSSCG00000040243*). These genes were upregulated in Iberian male fetuses and crossbred female fetuses. *COL9A2* and *COL9A1* are associated with body length, depth, and width in pigs [81]. This intriguing result should be investigated further.

Lysine demethylase 6A (*KDM6A*) showed higher expression in female than in male fetuses, independently of genotype. *KDM6A* is a lysine-specific histone H3 demethylase that controls tissue-specific expression of genes involved in development and the cell cycle [92–93]. In humans, *KDM6A* is an X-linked gene and haploinsufficiency is thought to cause hyperinsulinism in Turner syndrome [94]. However, we did not find any difference in plasma glucose levels between male and female fetuses. *KDM6A* is one of the known genes escaping X inactivation [95], which may explain its higher expression in females.

In the case of crossbreed male fetuses, expression of male-specific genes such as *EIF1AY* or *DDX3Y*, linked to chromosome Y, was found. These genes are located in the A-Zoospermia Factor (AZF) locus, which is associated with spermatogenesis [96]. AZF encodes the non-recombining region of the human Y chromosome, suggesting sex-specific expression of these genes. Interestingly, *EIF1AY* and *DDX3Y* are expressed in blood cells that encode human male-specific minor histocompatibility antigens. None of these antigens showed significant differential expression, but there was a trend towards significance (*p*< 0.001 and q = 0.09) in Iberian fetuses for *DDX3Y* gene.

Crossbred female fetuses exhibited downregulation of *DMTN*, *ALAS2*, *SLC4A1*, *AHSP* and *HBB*. Dematin (*DMTN*) helps maintain the cytoskeleton but in addition, disruption of dematin causes precipitous loss of erythrocyte membrane stability and severe hemolytic anemia [97]. *HBB* regulates nitric oxide biosynthesis, and its synthetase *ALAS2* was also downregulated in crossbred female fetuses. Mutations in *ALAS2* are known to cause X-linked sideroblastic anemia [98]. In support of this, downregulation of *AHSP* is related to erythrocyte differentiation, hemoglobin metabolic process, hemopoiesis, protein folding, and stabilization.

In agreement with these results, functional analysis of genes differentially expressed between crossbred female or male fetuses revealed pathways in *Tetrapyrrole Biosynthesis II* and *Heme Biosynthesis II*, which are critical for the oxygen-carrying capacity of erythrocytes. Functional analysis also predicted 138 biological functions and diseases affected by fetal sex (*p* < 0.01). Four of 5 molecules in the disease *Anemia* (*ALAS2*, *DMTN*, *HBB*, *SLC4A1* and *TUBB3*) were downregulated in crossbred female fetuses.

Regulator analysis identified 114 transcription factors that could be responsible for differential gene expression between crossbred male and female fetuses, with the transcription factors *GATA1* (*p* = 6.17E-08) and *KLF1* (*p* = 3.08E-04) being the most significant. The top regulatory transcription factors affected by fetal sex are shown in **Table 3**. *GATA1* is a master regulator that controls cellular growth and death [99] and regulates transcription of erythroid and megakaryocytic-expressed genes [100]. On the other hand, *KLF1* controls almost all aspects of erythroid cell development and maturation [101]. *GATA1* and *KLF1* independently regulate heme biosynthesis. Our results may mean that crossbred female fetuses have worse hematological system development and function than male fetuses.

**Table 3:** Top potential upstream regulators of transcriptional differences between Iberian LBW and HBW fetuses.

Upstream Regulator	Expression Log Ratio	Molecule Type	Predicted Activation State	Activation z-score*	*p*-value of overlap	Target molecules in dataset
**IRS2**		enzyme		0.243	3.28E-10	CEBPA,FOS,LPL,PCK1,THRSP
**NCOA2**		transcription regulator		1.067	2.72E-09	ADIPOQ,CEBPA,MMP13,PCK1,PLIN1
**N-cor**		group	Inhibited	-2.236	3.48E-08	ADIPOQ,CIDEC,MMP13,PCK1,PLIN1
**ADIPOQ**	1.364	other		-0.858	6.27E-08	ADIPOQ,CD163,CEBPA,LPL,PCK1
**IRS1**		enzyme		1.136	1.32E-07	ADIPOQ,CEBPA,FOS,LPL,PCK1
**LIPE**		enzyme		1.982	1.38E-07	ADIPOQ,CEBPA,FOS,PCK1,THRSP
**PPARA**		ligand-dependent nuclear receptor		0.242	1.51E-07	ADIPOQ,CEBPA,CIDEC,FOS,LPL,PCK1,PLIN1
**TNF**		cytokine		0.367	1.76E-06	ADIPOQ,CD163,CEBPA,FOS,LPL,MGP,MMP13,PLIN1
**SREBF1**		transcription regulator		0	2.02E-06	ADIPOQ,CEBPA,PCK1,PNPLA3,THRSP
**PPARG**		ligand-dependent nuclear receptor	Activated	2.223	2.47E-06	ADIPOQ,CEBPA,CIDEC,LPL,PCK1,PLIN1
**INS**		other		0.506	8.66E-06	CIDEC,FOS,LPL,PCK1
**SIRT1**		transcription regulator		-0.152	1.24E-05	ADIPOQ,MMP13,NELL2,PCK1,PNPLA3
**CEBPA**	0.844	transcription regulator			2.62E-05	ADIPOQ,CEBPA,DGAT2,FOS,PCK1
**NR3C1**		ligand-dependent nuclear receptor		1.033	3.23E-05	CEBPA,CIDEC,DGAT2,FOS,MMP13,PCK1
**ERK1/2**		group		-0.06	5.44E-05	ADIPOQ,CEBPA,FOS,MMP13
**P38 MAPK**		group		1.985	7.76E-05	CEBPA,FOS,MMP13,PCK1
**CIDEC**	1.884	other			4.09E-04	DGAT2,PLIN1
**IFNG**		cytokine		-1.187	1.06E-03	ADIPOQ,CD163,CEBPA,MMP13,PCK1
**MATN1**	2.718	other			1.07E-03	MATN4
**TGFB1**		growth factor		0.244	3.78E-03	ADIPOQ,CD163,FOS,MMP13
**FOS**	0.666	transcription regulator			4.96E-03	FOS,LPL,MMP13
**PLIN1**	1.33	other			1.60E-02	CIDEC
**LPL**	0.833	enzyme			3.69E-02	ADIPOQ
**TP53**		transcription regulator		-0.283	4.37E-02	CEBPA,FOS,MATN4,MMP13

* Indicates the activation state of predicted transcriptional regulators based on experimentally observed gene expression and existing literature. This allows prediction of the direction of regulation (activation or inhibition).

Female Iberian fetuses showed downregulation of genes associated with growth, such as *CNMD*, *COL9A2*, *COL9A1*, *MATN1*, and *MMP13*, as well as downregulation of fetuin-A (*AHSG*) and its paralog fetuin-B (*FETUB*). Both are adipokines/hepatokines secreted by the liver, but recent work suggests they are also co-expressed in placenta [102]. Increased fetuin-A levels are associated with insulin resistance in type 2 diabetes [103], but this effect seems to occur only in females [104]. Downregulation of fetuin-A and fetuin-B may be a strategy to protect against insulin resistance in female Iberian fetuses. Functional analysis identified 18 pathways affected by fetal sex in the Iberian genotype (**S12 Table**). The top canonical pathway was *FXR/RXR Activation* (*p* = 1.82E-06). The 4 DE genes in this canonical pathway (*TTR*, *TF*, *AHSG*, and *FETUB*) were downregulated in female Iberian fetuses. The farnesoid X receptor (FXR) is a member of the nuclear family of receptors and plays a crucial role in numerous metabolic processes. Along with retinol X receptor (RXR), FXR regulates bile acid levels in lipoprotein, lipid and glucose metabolism [105]. Activation of the FXR/RXR heterodimer by bile acids can decrease HDL and increase LDL plasma cholesterol levels, and decrease gluconeogenesis [105]. However, we did not observe any difference in cholesterol or glucose metabolism between male and female Iberian fetuses. We have previously reported that female Iberian fetuses have lower HDL levels and higher glucose levels than male Iberian fetuses at gestational day 62 [49]. These findings may mean that the *FXR/RXR Activation* pathway is affected by fetal sex, with preferential activation in males. Further studies with more individuals will be required to confirm these results.

Functional analysis identified 147 biological functions and diseases affected by fetal sex in the Iberian genotype (*p* < 0.01). Most of these were related to growth (*Connective Tissue Disorders*, *Developmental Disorder*, *Hereditary Disorder*, *Organismal Injury and Abnormalities*, *Skeletal and Muscular Disorders)*, consistent with the results of DE gene analysis.

Regulator analysis identified 119 transcription factors affected by fetal sex in the Iberian genotype. Of these, 10 were also identified as transcription factors in the crossbreed genotype (including *EPO* and *ITGA1*).

## Conclusion

Our findings show that genotype has a strong effect on the muscle transcriptome at a very early developmental stage and with an experimental design that reduced confounding maternal and environmental influences, but increases intra-group variability, due to selection of animals with extreme body weight values. We identified a large panel of transcriptional regulators that may drive the molecular pathways leading to developmental differences between genotypes. Genotype primarily affected biological functions and pathways related to muscle growth and lipid metabolism. Transcriptional differences between LBW and HBW individuals also differed between genotypes, with crossbred HBW animals showing upregulation of muscle development and survival-related functions, while Iberian HBW animals showed upregulation of lipid accumulation and adipogenesis-related functions. Although the sex effect was modest, our findings suggest that female crossbred fetuses have impaired hematological development and function and that female Iberian fetuses may employ a strategy to protect against insulin resistance.

## Supporting information

S1 Table Gene information, primer pairs and efficiency of genes used for quantitative real-time PCR validation. (XLSX)

S2 Table Correlation between RNA sequencing and quantitative PCR. (XLSX)

S3 Table Genes differentially expressed between Iberian and crossbred fetuses (q-value ≤ 0.05 and fold-change ≥ 1.3). (XLSX)

S4 Table Pathways enriched in genes differentially expressed between Iberian and crossbred fetuses (q-value ≤ 0.05 and fold-change ≥ 1.3). *Ratio*: number of differentially expressed (DE) genes in a pathway divided by the total number of genes in the same pathway. *z-score*: activation state of predicted transcriptional regulators based on experimentally observed gene expression and the literature. This allows prediction of the direction of activation (activation or inhibition).
(XLSX)

S5 Table Biological functions enriched in genes differentially expressed between Iberian and crossbred fetuses (p<0.0001). *Activation z-score*: Predicted activation status of biological function; the higher the value, the more activated the function is predicted to be. *Molecules*: number of molecules associated with the predicted biological function.
(XLSX)

S6 Table Potential upstream regulators affecting gene expression in Iberian and crossbred fetuses. *z-score: indicates the activation state of predicted transcriptional regulators based on experimentally observed gene expression and on the literature. This allows prediction of the direction of activation (activation or inhibition).
(XLSX)

S7 Table Genes differentially expressed between LBW and HBW fetuses with Iberian or crossbred genotypes (q-value ≤ 0.05 and fold-change ≥ 1.3). LBW = low body weight fetuses, HBW = high body weight fetuses, IBxIB = Iberian genotype, IBxLW = crossbred (Iberian x Large White).
(XLSX)

S8 Table Pathways enriched in genes differentially expressed between LBW and HBW fetuses with Iberian or crossbred genotypes (q-value ≤ 0.05 and fold-change ≥ 1.3). *Ratio*: number of differentially expressed (DE) genes in a pathway divided by the total number of genes in the same pathway. *Molecules*: number of molecules associated with the predicted biological function.
(XLSX)

S9 Table Biological functions enriched in genes differentially expressed between LBW and HBW crossbred fetuses (p-value < 0.01). *Activation z-score*: Predicted activation status of biological function; the higher the value, the more activated the function is predicted to be. *Molecules*: number of molecules associated with the predicted biological function.
(XLSX)

S10 Table Biological functions enriched in genes differentially expressed between LBW and HBW Iberian fetuses (p-value < 0.01). *Activation z-score*: Predicted activation status of biological function; the higher the value, the more activated the function is predicted to be. *Molecules*: number of molecules associated with the predicted biological function.
(XLSX)

S11 Table Genes differentially expressed between female and male Iberian and crossbred fetuses (q-value ≤ 0.05 and fold-change ≥ 1.3). (XLSX)

S12 Table Pathways enriched in genes diffferentially expressed between female and male fetuses with Iberian or crossbred genotypes (q-value ≤ 0.05 and fold-change ≥ 1.3). *Ratio*: number of differentially expressed (DE) genes in a pathway divided by the total number of genes in the same pathway.
(XLSX)
